# Effects of dose reduction on bone strength prediction using finite element analysis

**DOI:** 10.1038/srep38441

**Published:** 2016-12-09

**Authors:** D. Anitha, Karupppasamy Subburaj, Kai Mei, Felix K. Kopp, Peter Foehr, Peter B. Noel, Jan S. Kirschke, Thomas Baum

**Affiliations:** 1 Engineering Product Development (EPD) Pillar, Singapore University of Technology and Design (SUTD), 8 Somapah Road, 487372, Singapore; 2 Department of Radiology, Klinikum rechts der Isar, Technical University of Munich, Munich, Germany; 3 Department of Orthopaedics and Sports Orthopaedics, Biomechanical Laboratory, Klinikum rechts der Isar, Technical University of Munich, Munich, Germany; 4 Department of Neuroradiology, Klinikum rechts der Isar, Technical University of Munich, Muenchen, Germany

## Abstract

This study aimed to evaluate the effect of dose reduction, by means of tube exposure reduction, on bone strength prediction from finite-element (FE) analysis. Fresh thoracic mid-vertebrae specimens (n = 11) were imaged, using multi-detector computed tomography (MDCT), at different intensities of X-ray tube exposures (80, 150, 220 and 500 mAs). Bone mineral density (BMD) was estimated from the mid-slice of each specimen from MDCT images. Differences in image quality and geometry of each specimen were measured. FE analysis was performed on all specimens to predict fracture load. Paired t-tests were used to compare the results obtained, using the highest CT dose (500 mAs) as reference. Dose reduction had no significant impact on FE-predicted fracture loads, with significant correlations obtained with reference to 500 mAs, for 80 mAs (R^2^  = 0.997, p < 0.001), 150 mAs (R^2^ = 0.998, p < 0.001) and 220 mAs (R^2^ = 0.987, p < 0.001). There were no significant differences in volume quantification between the different doses examined. CT imaging radiation dose could be reduced substantially to 64% with no impact on strength estimates obtained from FE analysis. Reduced CT dose will enable early diagnosis and advanced monitoring of osteoporosis and associated fracture risk.

Incidence of fragility fractures, particularly in the hip and spine, is one of the most common skeletal pathologies, due to the increasing rate of growth of elderly population. It is an important public health problem that is predominantly attributed to the occurrence of osteoporosis. Osteoporosis is an age-related skeletal condition resulting in micro-architectural deterioration and loss of bone mass and density^1^. The pathological changes induced by osteoporosis remain often unnoticed until a fracture occurs. While osteoporosis can be a primary bone loss condition due to aging, recent studies have elucidated other underlying conditions such as multiple myeloma, diabetes, and cancer-treatments may elicit secondary osteoporosis^2^,^3^,^4^. Trauma, due to greater susceptibility to falls, is also a contributing cause to fractures in the elderly. Fractures lead to poor quality of life, immobility, dependency, morbidity, and significant healthcare costs^5^. Apart from being a socioeconomic burden, it also significantly impacts daily living and many patients do not return to their previous functional status^6^. In order to prevent the many sequelae of bone fragility, it is important to diagnose and treat the condition early to avoid significant health risk.

Dual energy X-ray absorptiometry (DXA) based bone mineral density (BMD) measurements in hip and spine (T- and Z- scores) have been used as the gold standard for the quantitative evaluation of bone loss. However, the lack of specificity and sensitivity renders BMD a less than reliable predictor of bone loss^1^. BMD has been demonstrated as an insufficient variable for bone strength prediction^7^ because there is a substantial overlap of BMD values of individuals with and without fractures^8^. Since the clinical outcome of bone loss is bone fracture, it is increasingly being recognized that the identification of patients at high risk of fracture is more important than the identification of people with bone loss^9^,^10^. Accurate assessment of fracture risk should ideally take into account other proven risk factors that add information to that provided by BMD alone^11^. The complex structure of bone tissue highlights the concept that its mechanical strength is derived from the interaction of different structural elements and needs to be accounted for while assessing fracture risk. According to several studies, BMD is only able to predict fractures with a detection rate of 30–50%^12^,^13^. This implies that BMD values often underestimate fracture risk, since osteoporotic fractures frequently occur in patients with non-pathological BMD values^14^. Thus, it is necessary to move beyond a singular dependence on BMD since it excludes underlying information on the local bone mass distribution.

Finite-element (FE) modeling has increasingly been used to perform structural analysis of bones to assess strength and understand the mechanism underlying bone fracture^15^,^16^. FE analysis acts as a non-invasive alternative to assess bone strength *in-vivo*, where radiological scan data of patients are translated into patient-specific three-dimensional (3D) anatomical models. These geometric models are provided with appropriate material properties and boundary value and loading conditions that aims to approximate the *in-vivo* fracture conditions as possible, to obtain realistic predictions of the structural strength and other related mechanical properties. Several studies have demonstrated the superiority of FE analysis based on computed tomography (CT) images to predict vertebral body compressive strength^17^,^18^,^19^. Also, patient-specific analysis and qualitative visualization of the vertebrae are other unique advantages of FE analysis. Results derived from FE analysis can provide clinicians with valuable information about bone quality for diagnosis, and targeted treatment planning and monitoring.

Although the use of CT for diagnostic imaging has increased dramatically over the years, the fear of high radiation exposure has prompted cautious use^20^. There is a general consensus that radiation dose that does not add value to patient care should be avoided. As such, there is little willingness to expose a patient to ionizing radiation to monitor skeletal conditions such as osteoporosis until it is too late when the patient has already sustained fractures. However, low-dose CT (LDCT) could be a feasible option for monitoring and evaluating bone health in osteoporotic patients and patients with predisposed skeletal conditions. Hence the need for clinical diagnostic evaluation for skeletal conditions emphasizes the importance of optimizing radiation dose of each individual CT examination and reducing radiation exposure due to CT^21^. Recently, a study examined the effect of radiation dose on image quality and mechanical strength of a single femur bone^22^. Another study by Musekyo *et al*. investigated the dose effect of CT-based volumetric BMD measurements^23^. In this study, we analyzed the dose-dependency of FE analysis, which has been shown to improve fracture risk prediction independent of vertebral volumetric BMD^24^ and examined the feasibility of using LDCT scans, instead of standard clinical imaging, to quantify bone loss in the vertebrae.

## Materials and Methods

### Study Specimens

Elven fresh thoracic mid-vertebral specimens from five human donors (two females aged 53 and 74 years and three males aged 46, 51, and 62 years, respectively), who have had no history of any skeletal diseases, were obtained from the Institute of Pathology and Anatomy, Munich. The mid-vertebral specimens as the middle third of each vertebra were cut out parallel to the endplates by using a band saw (FK 22, Bizerba, Balingen, Germany). The donors had dedicated their bodies for educational and research purposes to the local Institute prior to death, in compliance with local institutional and legislative requirements. The study was reviewed and approved by the local institutional review board (Ethikkommission der Fakultaet fuer Medizin der Technischen Universitaet Muenchen, Munich, Germany).

### MDCT Imaging

**Figure 1:**
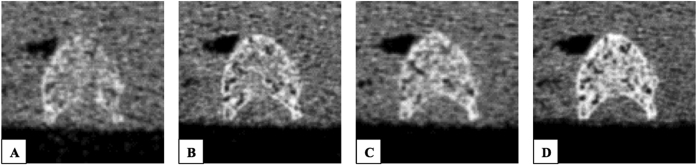
Images of the mid-slice of a typical mid-vertebra segment at different tube exposures, 80, 150, 220 and 500 mAs (A to D).

**Figure 2:**
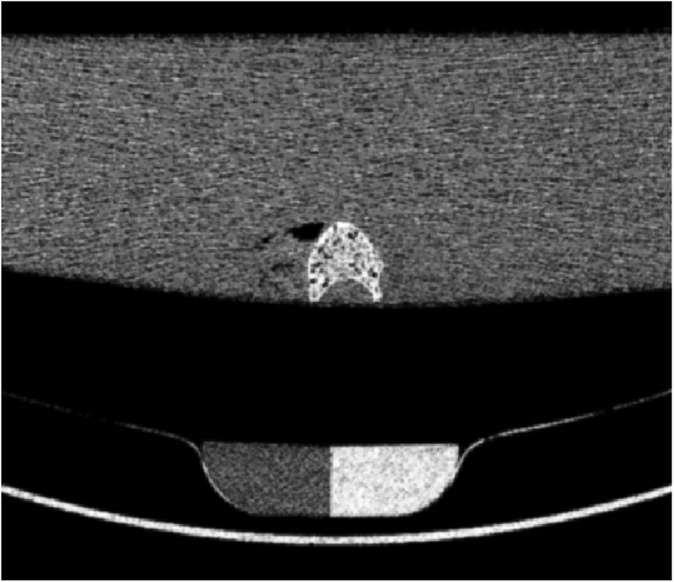
Representative image of a mid-vertebra segment depicting the bone-like and water-like phase of the reference phantom in the scanner mat beneath the water bath with a mid-vertebra segment.

MDCT imaging was performed with a 64-row MDCT scanner (SOMATOM Definition AS, Siemens Healthcare, Erlangen, Germany). The mid-vertebral specimens were scanned in a water bath to simulate the soft tissue environment. Four scans with different tube exposures (80, 150, 220, and 500 mAs) were performed (Fig. 1). All other scan parameters were kept constant: tube voltage of 120 kVp, image matrix of 512 × 512 pixels, pixel spacing of 0.44 × 0.44 mm^2^, and slice thickness of 0.4 mm. Transverse sections were reconstructed with a sharp reconstruction kernel (U75u). Mean dose estimate in terms of CTDIvol was 17.5, 32.9, 48.2 and 109.5 mGy for tube exposures 80, 150, 220 and 500 mAs respectively. For calibration purposes, a reference phantom with a bone-like and a water-like phase (Osteo Phantom, Siemens Healthcare) was placed in the scanner mat beneath patients (Fig. 2).

### Finite Element Modelling and Analysis

**Figure 3:**
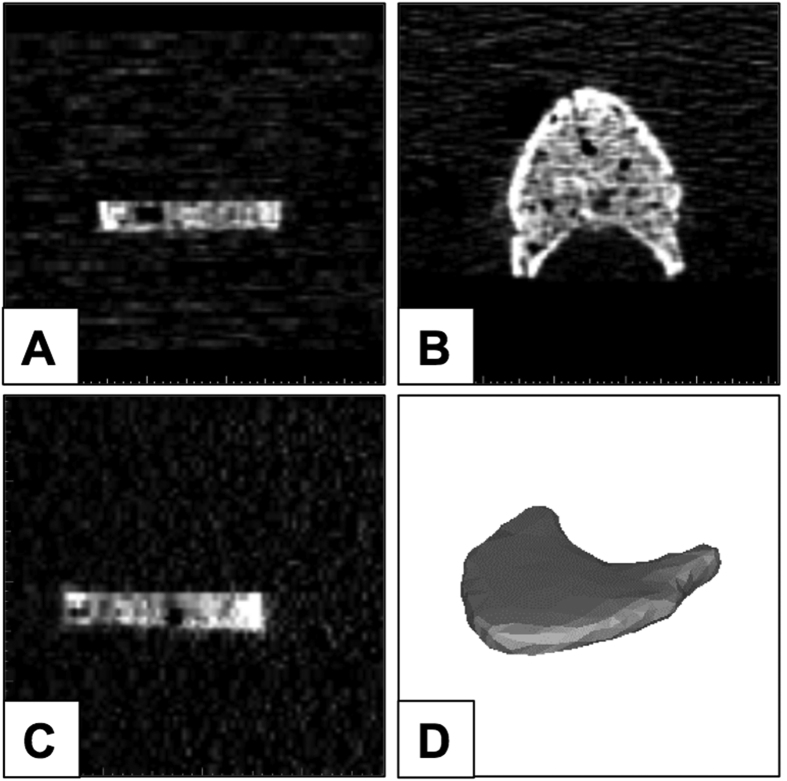
MDCT slice of one mid-vertebra specimen, coronal (**A**), axial (**B**), and sagittal (**C**) and the 3D rendered segment (**D**).

MDCT dataset of each specimen was imported and bone regions were segmented with commercial software Mimics (Materialise NV, Harislee, Belgium). The segmentation was performed semi-automatically, by applying HU values threshold as well as manual delineation of choosing the regions and contours (Fig. 3). Linear tetrahedral FE meshes were generated using 3-Matic (Materialise NV, Harislee, Belgium). Model convergence was investigated by differing the maximum edge lengths of mesh elements. The convergence study showed that a maximum edge length of 5 mm produced converging FE solutions and was thus used in the construction of the rest of the FE models. Material properties were mapped onto meshed models using Hounsfield unit (HU)-density and density-elastic modulus (E) relations *ρ*_*app*_ = *47* + 1.122*HU and *E* = *−349* + 5.82*ρ_*app*_ respectively, where E is expressed in MPa and the apparent density (ρ_app_) is expressed in g/cm^3^^25^. Transverse isotropy was assumed and the remaining engineering constants, including the Poisson’s ratio (v) and shear modulus (G) were assigned using the following relations: *E*_*xx*_ = *E*_*yy*_ = *0.333E*_*zz*_*, v*_*xy*_ = *0.381, v*_*xz*_ = *v*_*yz*_ = *0.104, G*_*xy*_ = *0.121E*_*zz*_ and *G*_*xz*_ = *G*_*yz*_ = *0.157E*_*zz*_. The ash density (ρ_ash_) was then derived from the ρ_app_ (*ρ*_*ash*_ = *ρ*_app_*0.6)^26^. Material strength (S) was defined for each material set based on its mean ρ_ash_ using correlations, as described previously^27^,^28^: *S* = *137ρ*_*ash*_^*1.88*^ for ρ_ash_ < 0.317 g/cm^3^ and *S* = *114ρ*_*ash*_^*1.72*^ for ρ_ash_ > 0.317 g/cm^3^. Material yield and ultimate failure were assumed to coincide and post-failure material behaviour was defined based on previously established relations, which have been shown to accurately predict strains^27^,^29^,^30^,^31^. Any negative modulus obtained was set to 0.0001 MPa^18^.

Boundary conditions were applied using ABAQUS version 6.10 (Hibbitt, Karlsson, and Sorensen, Inc., Pawtucket, RI, USA) to simulate axial compression, where the inferior surface was constrained in all directions and a displacement load condition was applied in the normal direction on the superior surface of each specimen. Fracture load was defined as the peak of the force-displacement curve, which was used as an estimate of bone strength in this study. The above-described FE method has been detailed and experimentally validated in our previous work^32^.

### MDCT-Geometry Analysis

The segmented bone regions of each specimen for each individual dose were converted to surface models and then compared in terms of Hausdorff distance, which is a measure of the distance between a point of one of two sets to a point of the other set^33^. For each surface model, in addition to the Hausdorff distance, the percentage of points that fall within 1 mm of the calculated distance were also estimated^22^, with the high CT dose (500 mAs) scans as the reference.

### MDCT-based BMD Analysis

Elliptical regions of interest (ROIs) were placed on an axial mid-slice for each specimen. These ROIS automatically provide the mean density in terms of HUs and its standard deviation within the region. Reference ROIs were placed within bone density calibration phantoms composed of hydroxyapatite (HA), where the water-like part of phantom has HA density of 0 mg/cm^3^ (HA_w_) and the bone-like part of the phantom has HA density of 200 mg/cm^3^ (HA_b_). BMD was calculated by the following formula: BMD = [HA_b_/ (HU_b_ – HU_w_)]*(HU-HU_w_)^34^, by assuming linear interpolation between the HUs of the water-like regions (HU_w_) and bone-like regions (HU_b_).

### Statistical Analysis

A paired two-tailed t-test was used to compare the differences in image quality, geometry, and FE-predicted fracture loads between CT doses (80, 150 and 220 mAs). Regression equations and the coefficients of determination (R^2^) were used to evaluate the linear correlations between fracture loads obtained for individual doses as well as between MDCT-determined BMD values and fracture loads. High dose CT (500 mAs) scans were taken as the reference for comparisons between individual doses. The reproducibility of the fracture loads for each individual dose (80, 150 and 220 mAs), with reference to the fracture loads obtained at 500 mAs, were assessed using the Bland and Altman plots^35^ and Lin’s concordance correlation coefficient (r_c_)^36^. All analyses were performed using spreadsheet application (Microsoft Office Excel 2010, Redmond, WA), except for Lin’s coefficient, which was calculated using MedCalc version 16.8.4 (MedCalc Software, Mariakerke, Belgium). A value of p < 0.05 was considered as statistically significant.

## Results

### Image quality

**Figure 4:**
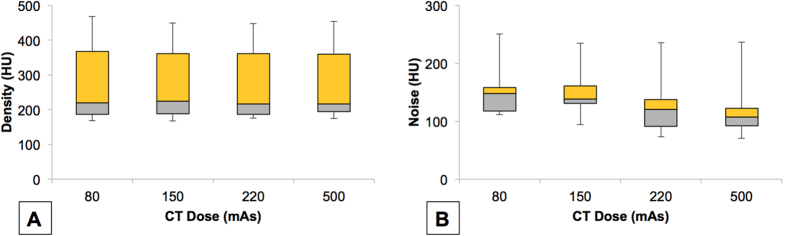
Box plots showing the differences in density and noise between the four CT doses (80, 150, 220 and 500 mAs). The horizontal lines within the boxes represent the median, while the top and bottom lines of the boxes represent the first and third quartiles respectively. The whiskers represent the minimum and maximum values.

The differences between each CT dose in terms of the density and noise were analyzed. The mean density, mean noise and their respective standard deviations were tabulated (Table 1). There were no significant differences in mean density between CT doses (Fig. 4A). However, image noise was significantly lower in 500 mAs, compared to 80 mAs (25.3%; p < 0.001) (Fig. 4B). There were no significant differences observed in mean noise with 150 mAs and 220 mAs, as compared to 500 mAs.

### FE-predicted Fracture Load

**Figure 5:**
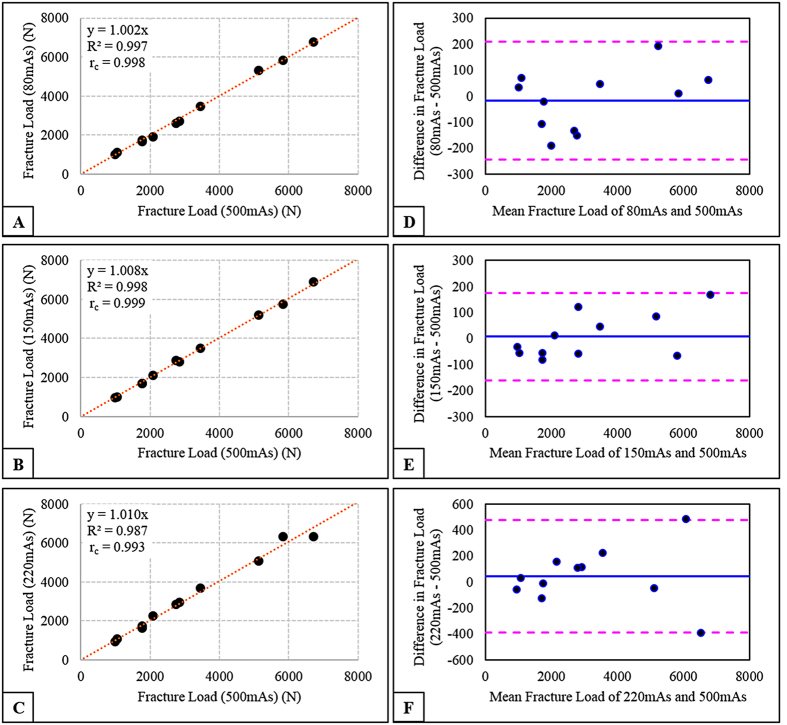
Correlation between fracture load values for each individual dose, 80 mAs (**A**), 150 mAs (**B**) & 220 mAs (**C**) as a function of fracture load values obtained at 500 mAs, and Bland-Altman plots depicting the mean of each individual dose (80, 150, 200 mAs) and of 500 mAs versus the difference between the two doses (**D–F**). The horizontal lines indicate the mean value, and 95% confidence intervals at ±1.96 standard deviation (SD).

**Figure 6:**
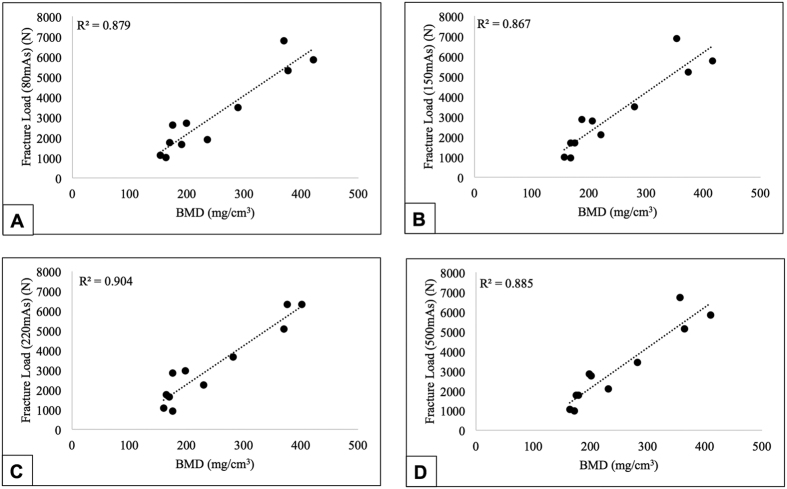
Correlation between BMD and fracture load values for each individual dose, 80 mAs (**A**), 150 mAs (**B**), 220 mAs (**C**) & 500 mAs (**D**).

Significant correlations were obtained for FE-predicted fracture load values for 80 mAs (R^2^ = 0.997, p < 0.001), 150 mAs (R^2^ = 0.998, p < 0.001) and 220 mAs (R^2^ = 0.987, p < 0.001), against 500 mAs as the reference (Fig. 5A-C). There were no significant differences in fracture loads obtained for each individual dose against 500 mAs. Good concordance between each individual dose and 500 mAs was also found for 80 mAs (r_c_ = 0.998), 150 mAs (r_c_ = 0.999) and 220 mAs (r_c_ = 0.993) (Fig. 5D-F). However, there were no significant correlations in the Bland–Altman plots for either dose (80, 150 and 220 mAs), indicating there was a consistent bias. Also, significant correlations were observed between MDCT-determined BMD and fracture load values for 80 mAs (R^2^ = 0.879, p < 0.001), 150 mAs (R^2^ = 0.867, p < 0.001), 220 mAs (R^2^ = 0.904, p < 0.001), and 500 mAs (R^2^ = 0.885, p < 0.001) (Fig. 6). FE-predicted strength estimates for each mid-vertebra specimen across different doses (80, 150, 220 and 500 mAs) are listed in Supplementary Table 1.

### Geometry

Comparing the surface models of 80, 150, and 220 mAs against 500 mAs, the maximum Hausdorff distance obtained was approximately the same (Table 2) for all doses and no significant differences were observed. Similarly, the percentage of points that fell within a 1 mm distance was about 90%. In addition, the mean error in volume decreased with CT dose, where mean error in volume was approximately 7%, 4%, and 2% for the 80 mAs, 150 mAs, and 220 mAs respectively. There were no significant differences between individual doses for the volumes of models generated for FE analysis.

## 

## Discussion

In this study, FE-predicted fracture load values obtained from MDCT data were not affected by tube exposure reduction from 500 to 80 mAs, with no significant differences in terms of correlation, with the high CT dose (500 mAs) scans used as reference. Although the noise significantly reduced from LDCT (80 mAs) to high-dose CT (500 mAs) specimens, there were no differences in outcome, in terms of fracture load values obtained from FE analysis, since the density values remained unaffected. Accordingly, there were no significant differences observed in volume segmentations between the doses examined in this study. The reliability of the fracture load values were further emphasized with significant correlations obtained with MDCT-determined BMD values, since several studies have also established a linear correlation^37^. These findings are consistent with the only one other published study that the authors are aware of, which compared CT-based FE analysis of a single femur bone with varying radiation doses from 180 mAs to 80 mAs^22^.

In developed countries, the use of CT is known to account for almost 17% of all radiological examinations, but is attributed to 47% or higher of medical radiation dose^38^. With such an alarming contribution, it is necessary to evaluate the efficacy of routine CT imaging. In patients with predisposed skeletal conditions, routine CT imaging to evaluate bone strength is always performed with caution. In addition, due to the high ionizing radiation exposure of routine imaging, follow-up scans are limited until or unless necessary. As such, the evaluation of bone strength may valuable in early diagnosis of skeletal conditions such as osteoporosis. Not only does LDCT FE analysis provide the possibility of scanning in patients with predisposed conditions, but also allows more frequent follow-up scans, which implies that bone strength can be predicted and treated early as well as monitored regularly. Furthermore, risk for bone fracture can be more accurately diagnosed with the complementation of FE-predicted bone strength information, in addition to BMD. The aim of this study was to examine whether LDCT examinations (80 mAs and 150 mAs) were as accurate, in comparison to standard routine CT scans (220 mAs) and high dose CT scans (500 mAs) in providing structural information using FE analysis.

The main implication of our findings is that dose reduction, with respects to tube exposure, does not affect structural findings, such as strength estimations, obtained from engineering analysis. This is critical because a dose reduction from routine clinical imaging dose (220 mAs) to LDCT dose (80 mAs) is a significant reduction by 64% but the highest percentage error in volume segmentations did not exceed 10%, which certainly justifies its consideration as a clinical tool in the biomechanical quantification of bone strength. Based on concerns of inferior image quality and diagnostic accuracy, LDCT has still not been clinically appreciated for assessment of patients with bone loss. These findings support that LDCT may be utilized as the preliminary imaging examination, to accurately evaluate bone strength and the consequent fracture risk of patients.

Several studies have demonstrated the usefulness of LDCT for clinical diagnosis in patients with other pathological skeletal conditions. For instance, Horger *et al*. found low-dose MDCT scans (tube exposure: 40–60 mAs) were capable of diagnosing lytic bone changes and evaluating fracture risk, even in patients with known or expected reduced bone density, in patients with multiple myeloma^39^. Bohy *et al*. showed that a 65% reduction in CT dose does not impact diagnostic findings evaluated in patients with lumbar disk herniation^40^. Similarly, Mulkens *et al*. demonstrated that a 61–71% dose reduction can be obtained with negligible reduction in image quality^41^ in the assessment of cervical spine clearance in patients with blunt force trauma. In line with these findings, our results show that a 64% dose reduction in CT dose did not affect image quality as well as bone strength predictions. Hence, the first step may be to opt for LDCT scanning protocol, accompanied with structural engineering analysis, which bears several benefits in terms of assessing bone quality and predicting fracture risk.

A key benefit of a combined LDCT and structural engineering analysis protocol is that the outcome of improved diagnosis of osteoporosis. As aforementioned, BMD alone is not sufficient enough to describe the intricacies involving bone architecture, geometric, and structural properties. The unique feature of FE analysis is the integration of structure, geometry and density distribution to evaluate bone strength^42^. As such, bone strength assessment via FE-analysis using LDCT scans can be a complementing measure to patients suffering from bone loss. Since BMD is insufficient to accurately predict bone strength, LDCT leads the possibility of diagnosing bone loss with more predictive components of fracture risk. Furthermore, with LDCT scanning, there is a true possibility of increasing frequency of follow up scans. This means that apart from diagnosing bone loss, monitoring bone loss and effect of treatment at shorter intervals will be plausible. Another key benefit of LDCT is the increased feasibility of radiological examinations in patients predisposed to other pathological skeletal conditions. It has been shown that patients undergoing cancer treatments, in the form of radiation therapy, experience insufficiency fractures^43^. As such, subjecting them to additional CT-based radiation for diagnostic evaluation of bone loss may not be practical, because of a probable risk of radiation-induced malignancy^44^ or fear of aggravated bone loss, leading to secondary bone fractures. Given these potential risks, it can be easily argued that radiation exposure associated with routine CT imaging for the evaluation of bone loss is inapplicable.

There are several limitations inherent to this study. The main limitation is the lack of verification on the use of LDCT in evaluating micro-architectural variables, i.e. trabecular-level changes, which may produce different results. However, the accuracy and reliability of FE-predicted bone strength have been demonstrated in several studies^16^,^29^,^45^. Also, this study only considered CT dose reduction via the variation of tube current but dose can also be reduced by reducing tube voltage or increasing pitch^38^ for instance. Nevertheless, variation of tube current is the most prevalent method for CT dose reduction^22^. In addition, a snap sample number of n = 11 were used in this study. The use of a larger sample size will provide statistical weighting to the findings obtained. However, despite these limitations, the merit of these findings is that LDCT, in combination with FE analysis, may be more valuable than we realize as a clinical diagnostic tool.

In conclusion, our results demonstrated that CT dose reduction by up to 64% does not affect strength estimates obtained from FE analysis. One of the clinical questions that should be answered is: is routine imaging required? If there is a clear indication for imaging, the option of LDCT scans should first be considered. Clinical diagnostic evaluation, in terms of radiological examinations, has profound impact on the overall radiation dose delivered to each individual. Apart from diagnosis of bone loss, efforts should focus onto monitoring bone loss to predict fractures early and therefore, CT scanning protocol should be optimized for the intended clinical evaluation.

## Additional Information

**How to cite this article**: Anitha, D. *et al*. Effects of dose reduction on bone strength prediction using finite element analysis. *Sci. Rep.* **6**, 38441; doi: 10.1038/srep38441 (2016).

**Publisher's note:** Springer Nature remains neutral with regard to jurisdictional claims in published maps and institutional affiliations.

## Supplementary Material

Supplementary Table

## Figures and Tables

**Table 1:** Mean density and noise and their respective standard deviations (SD) obtained for each CT dose.

Mean	CT Dose (mAs)			
	80	150	220	500
Density (SD)	272 (34)	270 (30)	269 (32)	273 (31)
Noise (SD)	152 (13)	148 (11)	127 (14)	121 (13)

**Table 2:** Hausdorff distance, percentage of points within 1 mm of Hausdorff distance and error in volumes obtained, with 500 mAs as reference.

Mean	CT Dose (mAs)		
	80	150	220
Hausdorff Distance (SD) (mm)	3.57 (0.20)	3.45 (0.24)	3.34 (0.14)
Points < 1 mm (%)	90.7 (1.5)	92.0 (0.8)	90.4 (1.0)
Error in Volume (%)	7.02 (1.73)	4.26 (1.13)	2.03 (0.45)
