# Supplementary material for: Effects of dose reduction on bone strength prediction using finite element analysis
Source: Sci Rep. 2016 Dec 9;6:38441. doi: 10.1038/srep38441 (PMC5146932; doi:10.1038/srep38441)
Supplement: Supplementary Table [file srep38441-s1.pdf]

Supplementary Information

Effects of dose reduction on bone strength prediction using finite element analysis

D Anitha[1], Karupppasamy Subburaj[1], Kai Mei[2], Felix K. Kopp[2], Peter Foehr[3], Peter B. Noel[2], Jan S. Kirschke[4], Thomas Baum[2]

[1] Engineering Product Development (EPD) Pillar, Singapore University of Technology and Design (SUTD), 8 Somapah Road, Singapore 487372

[2] Department of Radiology, Klinikum rechts der Isar, Technical University of Munich, Munich, Germany

[3] Department of Orthopaedics and Sports Orthopaedics, Biomechanical Laboratory, Klinikum rechts der Isar, Technical University of Munich, Munich, Germany

[4] Department of Neuroradiology, Klinikum rechts der Isar, Technical University of Munich, Muenchen, Germany

*Corresponding author: Subburaj Karupppasamy, subburaj@sutd.edu.sg

Supplementary Table S1. FE-predicted strength estimates for each mid-vertebra specimen across different doses (80, 150, 220 and 500 mAs)

Subject	Sex	Age	Vertebra	Fracture load (N) / Dose (mAs)			
				80 mAs	150	220	500
1	male	51	T5	2701	2795	2967	2853
1	male	51	T6	6782	6888	6328	6720
2	female	53	T7	1118	993	1081	1049
2	female	53	T10	2615	2868	2857	2747
3	female	74	T6	1656	1706	1751	1764
3	female	74	T7	5320	5214	5080	5129
3	female	74	T8	1751	1691	1647	1773
3	female	74	T9	5844	5769	6319	5835
4	male	46	T8	3487	3488	3664	3442
4	male	46	T10	1898	2098	2244	2088
5	male	62	T10	1018	952	925	985
